# The coffee-machine bacteriome: biodiversity and colonisation of the wasted coffee tray leach

**DOI:** 10.1038/srep17163

**Published:** 2015-11-23

**Authors:** Cristina Vilanova, Alba Iglesias, Manuel Porcar

**Affiliations:** 1 Universitat de València (Cavanilles Institute of Biodiversity and Evolutionary Biology), 46020 Valencia, Spain; 2 Fundació General de la Universitat de València, Spain

## Abstract

Microbial communities are ubiquitous in both natural and artificial environments. However, microbial diversity is usually reduced under strong selection pressures, such as those present in habitats rich in recalcitrant or toxic compounds displaying antimicrobial properties. Caffeine is a natural alkaloid present in coffee, tea and soft drinks with well-known antibacterial properties. Here we present the first systematic analysis of coffee machine-associated bacteria. We sampled the coffee waste reservoir of ten different Nespresso machines and conducted a dynamic monitoring of the colonization process in a new machine. Our results reveal the existence of a varied bacterial community in all the machines sampled, and a rapid colonisation process of the coffee leach. The community developed from a pioneering pool of enterobacteria and other opportunistic taxa to a mature but still highly variable microbiome rich in coffee-adapted bacteria. The bacterial communities described here, for the first time, are potential drivers of biotechnologically relevant processes including decaffeination and bioremediation.

Caffeine (1,3,7-trimethylxanthine) is a natural alkaloid with anti-herbivorous properties produced by *Coffea arabica* and *Coffea canephora*, which is present in a wide range of beverages including coffee, tea and soft –mainly cola- drinks. Caffeine is a well-known bioactive compound with stimulating effects on the central nervous system, along with a range of other potentially positive effects on human health. Such effects range from enhancing long-term memory^1^, improving sports performance^2^, inactivating breast cancer-associated myofibroblasts^3^, reducing the risk of type 2 diabetes mellitus^4^, or even reducing the risk of mortality among coffee consumers^5^. However, it should also be noted that caffeine intake habits are often linked to living styles and thus it is difficult to draw epidemiological conclusions linking caffeine intake to health.

Caffeine may be an environmental pollutant^6^, and has also been proposed as an easily detectable marker for untreated wastewater^7^. Indeed, the presence of caffeine in the natural environment is one of the best indicators of anthropogenic contamination. Caffeine bioactivity on human health and the environment has led to the development of processes to remove caffeine, either to yield decaffeinated products or to degrade environmental caffeine. Decaffeination, namely the caffeine removal process, is used industrially to produce low-caffeine beverages and can also be implemented for environmental remediation. One intriguing option is to use microorganisms to perform decaffeination processes. Some microorganisms have been reported to degrade caffeine, such as *Aspergillus tamarii*^8^, *Trichosporon asahii*^9^, *Pseudomonas* sp.^10^,^11^ or *P. putida*^12^.

In this work, we report a diversity analysis aiming to characterise bacterial communities growing on coffee leach waste, using high througput sequencing, culturing, and electron microscocopy techniques. To achieve this goal, we have chosen one of the most widespread coffee preparation systems, Nespresso, due to its popularity and standard nature. In fact, Nespresso-compatible machines are highly standardized coffee making devices (same capsule type, same basic design, same pressure: 19 bars), and they represent a unique oportunity for a massive biological screening. Here we present the first attempt to do so. We have sampled the inner drip tray below the capsule container, in which coffee lyxiviate accumulates. We have analysed ten domestic and semi-domestic machines and studied the dynamic colonization process in a brand new Inissia Krups machine operated in our laboratory. This is the first systematic analysis of the microbial diversity associated to coffee machines. Our results may shed light on the microbial arsenal of caffeine degraders with important implications for both medicine and biotechnology.

## Results and Discussion

**Figure 1:**
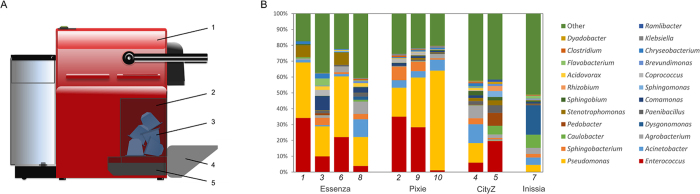
(**A**) Schematic representation of a Nespresso machine (1) including a capsule (3) container (2), cup tray (4) and a drip tray (5), which was sampled in this work. (**B**) Bacterial profile of the nine Nespresso machines sampled according to 16S rRNA gene sequencing. Samples numbered in accordance to Table 1.

The waste coffee in the capsule container of nine different Nespresso machines operated for at least one year was sampled (Fig. 1A). In one case (CityZ model), the cup tray was also sampled independently as it does not connect with the capsule container. The high throughput sequencing and analysis of the 16S rRNA gene amplicons from all the machines revealed a significant bacterial diversity, with the total number of identified genera ranging from 35 to 67. Although relatively similar microbial profiles were detected, there was an important variation in the frequency of particular taxa. *Enterococcus* sp. and *Pseudomonas* sp. proved to be the main taxa as they were moderately to highly abundant in nine out of the ten samples analysed. Other frequent genera were *Stenotrophomonas, Sphingobacterium, Acinetobacter* and, to a lesser extent, *Coprococcus, Paenibacillus* or *Agrobacterium*. *Dysgomonas* was very frequent in the Inissia machine, accounting for 15% of the sequences (Fig. 1B). No differences were detected between machine models (Table 1) or use (domestic *vs.* communal).

One of the two most frequent genera found in the coffee machines was *Pseudomonas*, which is also one of the few reported examples of a caffeine-degrading bacterium. Indeed, *Pseudomonas* sp. has been known to catabolise caffeine since the seventies^13^, and is reported to degrade up to 15 g/L of caffeine through an N- demethylation reaction, which along with C-8 oxydation represent the two potential catabolic pathways^14^. Species reported to display caffeine degradation abilities are *P. alcaligenes*^15^ and *P. putida* (strains C1, CBB1 or CBB5). In fact, *P. putida* N-demethylation genes have been used to genetically engineer a caffeine “addicted” version of *E. coli*^16^, and caffeine removal from sewage by bioremediation with *P. putida* has also been proposed^17^.

The abundance of *Enterococcus* spp. in caffeine-rich leach might not necessarily involve unreported caffeine degradation abilities in *Enterococcus*, but it might simply be a consequence of tolerance to certain caffeine levels. The same applies to other frequent taxa. Interestingly, this genus has previously been associated with coffee^18^, along with several others detected in this work. For example, *Acinetobacter* sp. has been isolated during coffee fermentation^19^, while *Stenotrophomonas* sp., *Curtobacterium* sp., and *Pseudomonas* sp. are abundant in the coffee seed^20^.

**Figure 2:**
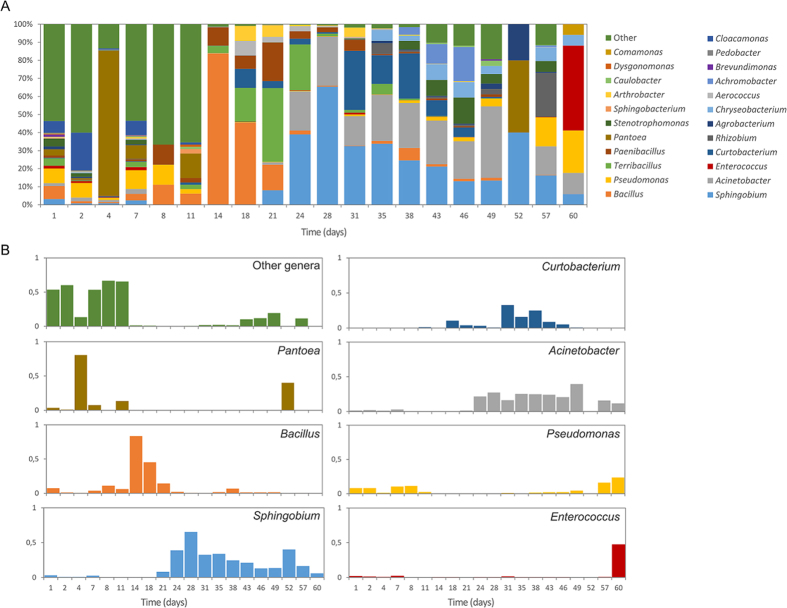
Bacterial colonisation in a brand new Nespresso Krups Inissia machine. (**A**) Bacterial profile in the drip tray during the two months of operation according to 16S rRNA gene monitoring. (**B**) Ecological succession of the main taxa during the experiment, represented as the variation of their relative frequencies.

**Figure 3:**
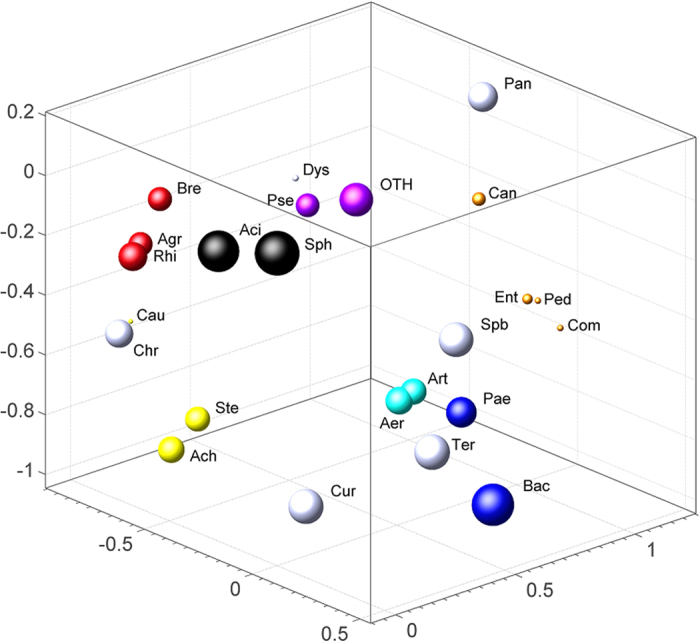
Correlations among the bacterial genera detected in this work. Distances correspond to the linear statistical correlation. Sizes of the spheres are proportional to the relative abundances in logarithmic scale. Highly correlated genera are shown in the same colour. Sph, *Sphingobium*; Bac, *Bacillus*; Aci, *Acinetobacter*; Ter, *Terribacillus*; Cur, *Curtobacterium*; Pae, *Paenibacillus*; Pan, *Pantoea*; Rhi, *Rhizobium*; Chr, *Chryseobacterium*; Aer, *Aerococcus*; Art, *Arthrobacter*; Ste, *Stenotrophomonas*; Ach, *Achromobacter*; Pse, *Pseudomonas*; Can, Candidatus *Cloacamonas*; Agr, *Agrobacterium*; Bre, *Brevundimonas*; Ent, *Enterococcus*; Cau, *Caulobacter*; Dys, *Dysgonomonas*; Spb, *Sphingobacterium*; Ped, *Pedobacter*; Com, *Comamonas*; Oth, Other genera.

The colonisation process of the wasted coffee leach was studied in an experiment using a brand new Krups Inissia machine (located in a separate room within our laboratory). The experiment lasted two months, during which leach samples were taken and bacterial diversity analysed, with a significant variation in the taxonomic profiles detected. The initially high species richness was substituted by a relatively simpler, but still highly variable, species composition (species richness significantly dropped 14 days after the beginning of the experiment; t-test p-value = 0.039). During the first 11–13 days, *Pantoea* sp., *Cloacomonas* sp. and, to a lesser extent, *Brevundimonas* sp. were relatively abundant but amounts decreased to undetectable levels by the end of the experiment. All these taxa were largely substituted by *Pseudomonas* sp.*, Acinetobacter* sp., and *Sphingobium* species, which reached a peak and then fluctuated (*Sphingobium* sp., *Bacillus* sp.) or reached the highest levels at the end of the experiment (*Pseudomonas* sp.*, Acinetobacter* sp.) as shown in Fig. 2A. The first 30 days exhibited greater instability in the bacterial communities, as deduced by the consecutive peaks of very abundant taxa, which were substituted by a more balanced bacterial composition after one month. As in other studies on different environments^21^,^22^, these results strongly suggest a long ecological succession during the first month, in which generalist bacterial taxa, including enterobacteriaceae genera such as *Pantoea*, are the first colonizers but are then displaced by successive waves of other taxa. The main keyplayers observed during this succession were, in order (Fig. 2B): enterobacteria (genus *Pantoea*; peaking 4–11 days after the beginning of the experiment), Firmicutes (three genera of the bacillaceae family: *Bacillus, Terribacillus, Paenibacillus*; peaking after 14–21 days); and, finally, the sphingomonadales genus *Sphingobium* (proteobacteria), the actinomycetales genus *Curtobacterium* (actinobacteria), and the pseudomonadales genus *Acinetobacter* (proteobacteria), peaking after 28, 31 and 49 days, respectively. These taxa gave way to a different bacterial profile dominated by *Pseudomonas* sp. and *Enterococcus* sp. after two months of the experiment. This profile was very similar to that found in the nine other coffee machines sampled (Fig. 1B) which had been operated for a longer time, suggesting that the particular physico-chemical conditions (cycles of high temperature, constant caffeine accumulation, etc.) of coffee leach, rather than the influence of the user or the number of uses, are the main force shaping the composition of the microbial community. A mathematical modelling performed on the dynamic series of 16S rRNA gene data revealed statistically significant correlations among the detected taxa, indicating that the distribution of bacterial genera in time is not random (Fig. 3).

Most of the taxa we identified during the colonisation process of the coffee machine operated in our laboratory have previously been found in natural coffee-related environments. Species belonging to the genera *Acinetobacter* and *Bacillus*, and also some enterobacteria, have been detected during the natural fermentation of coffee beans^23^,^19^, whereas *Paenibacillus* and other Bacteroidetes and Firmicutes species have proved abundant in the composting process of coffee hulls^24^,^25^. Despite some reports describing the ability of different *Sphingobium* species to degrade toxic molecules, such as bisphenols^26^ and hydroquinones^27^, this is the first report where *Sphingobium* sp. has been associated to a caffeine-rich environment.

**Figure 4:**
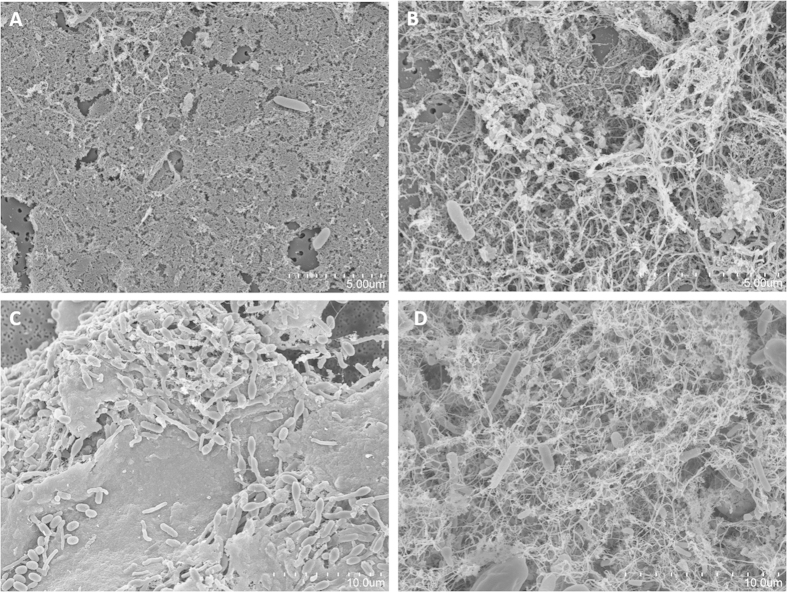
Chronological series of SEM images of the drip tray samples taken after 4 (**A**), 8 (**B**), 14 (**C**), and 21 days (**D**) of operation. Figure C corresponds to a sample highly abundant in *Bacillus* spp. Scale bars are indicated in each case.

In addition to the 16S rRNA gene monitoring, we followed up changes in the coffee leach microbial diversity through scanning electron microscopy (SEM). Figure 4 shows a dynamic series of samples taken at different time points (4, 8, 14, and 21 days after the first day of operation) during the first month. Microbial biomass increased throughout the analysis, and variations in the composition and viscosity of the coffee leach were also evident. For example, a filamentous matrix was observed at days 8 and 21 (Fig. 4). At day 14, the sample was dominated by a single shape of bacterial cells, which interestingly coincided with an overwhelming relative abundance of *Bacillus* spp. in that sample (Fig. 2A). Further experiments are needed to determine whether microbial community changes are the cause or the effect of the variations in the composition of coffee leach as shown by SEM.

Our results show, for the first time, that coffee leach from standard capsule machines is a rich substrate for bacterial growth; that caffeine content does not prevent a rich bacterial biodiversity from rapidly colonising coffee leach; and that microbial succession from an initial pool of generalist bacteria gives way to an apparently coffee-adapted but still highly variable bacteriome. This bacteriome is rich in species previously reported to be associated with the coffee plant and/or the coffee fermentation processes. Colonising bacteria might be of environmental origin (no cultivable microorganisms nor bacterial DNA was detected in coffee capsules, data not shown), whereas heterogeneity of bacterial composition may relate to factors such as cleaning habits and, specially, the frequency of machine use (with higher frequencies presumably correlating with increased volume and temperature of the coffee leach). Further studies comprising more coffee machines, deep genome sequencing of the microbial communities therein, and even functional metagenomics, are required to contribute to shed light on the microbial ecology of coffee leach in capsule machines.

The presence of bacterial genera with pathogenic properties and the fast recovery of the communities after rinsing the capsule container, strongly suggest the need for frequent maintenance of the capsule container of these machines. Maintenance should employ bacteriostatic compounds, and avoid contact of the coffee leach with other parts of the machine to avoid unintended contamination of the beverage. On the other hand, the resistant microbial communities we describe here (microbial consortia, individual caffeine degrading/tolerant species or as a source of metabolic pathways and genes) may represent a promising tool for biological coffee decaffeination processes and for environmental caffeine decontamination.

## Methods

### Sampling

Nine Nespresso machines, which had been operated for at least one year, used either at home (domestic) or in academic departments, institutes or biotechnology companies (communal) in the Valencia (Spain) area were aseptically sampled (Table 1). The coffee lixiviate from wasted capsules present in the drip tray placed below the capsule container was sampled using a sterile Pasteur pipette. In one case (CityZ), the small space under the drip grid and cup support facilities was sampled and treated separately (Fig. 1A; Table 1). In all cases, the average temperature of the room where the coffee machines were operated was close to 25 °C.

Additionally to these machines, a Krups Inissia machine was purchased for the present work and normally operated in our laboratory for five months with a daily use of around 1–5 capsules per day. Sampling was performed at increasing time lapses and consisted of the removal of most (except 5 mL, approximately) of the lixiviated liquid. When the volume of the lixiviate was smaller than 5 mL, no samples were taken. All the detachable pieces (drip grid and cup support, drip tray and capsule container were thoroughly rinsed once (day 28) with tap water.

In all cases, 2 mL aliquots of the samples were immediately used for DNA isolation, whereas the remaining volume was stored at −80 °C.

### Scanning electron microscopy

Aliquots from each sample were filtered through a 0.2 micrometre filter (Corning Inc.), fixed with a 2% paraformalin - 2.5% glutaraldehyde solution, and lightly washed with filtered-sterile phosphate buffer (PBS). Then, small pieces of the filters were placed inside microporous capsules (30 μm pore size, available from Ted Pella Inc. product number 4619) and subjected to successive dehydration steps in growing ethanol solutions up to 100% ethanol, following critical point drying in an Autosamdri 814 (Tousimis). These fragments were then placed on SEM stubs with silver conducting paint TAAB S269 and examined under a scanning electron microscope Hitachi S-4100.

### DNA isolation and PCR amplification

Two mL aliquots of each sample were centrifuged at 11,000 g for 3 min and the resulting pellets were washed twice with sterile PBS buffer (NaCl 8 g/L, KCl 0.2 g/L, Na_2_HPO_4_ 1.44 g/L, KH_2_PO_4_ 0.24 g/L, pH adjusted to 7.4). A widely-used DNA isolation protocol based on lysozyme treatment and alkaline lysis, suitable for the treatment of both Gram negative and Gram positive bacteria, was used to obtain metagenomic DNA. Briefly, each sample was treated with 2 mg/mL lysozyme (30 min, 37 °C) to ensure the lysis of Gram positive bacteria, and DNA was isolated with an alkaline solution (Tris 300 mM, SDS 1.25%, sucrose 5%, EDTA 10 mM; adjusted to pH 8.0) followed by precipitation with 3M potassium acetate (pH 5.0) and isopropanol. The quality of the DNA was checked on a 0.8% (w/v) agarose gel and quantified with Nanodrop-1000 Spectrophotometer (Thermo Scientific, Wilmington, DE). Universal primer sequences 28F (5′-GAG TTT GAT CNT GGC TCA G-3′) and 519R (5′-GTN TTA CNG CGG CKG CTG-3′), were chosen for the PCR-amplification of the 16S ribosomal RNA gene since they targeted the V1-V3 hypervariable region (commonly analysed in metagenomic studies) and produced an amplicon of a suitable length (500 bp) for the subsequent sequencing library construction. A short (9–11 nucleotides) barcode sequence followed by a four-nucleotide spacer (CGAT) was included at the 5′ end of the oligonucleotides used as forward primers to enable sequence assignment to samples after high-throughput sequencing. All the amplifications were performed under the following conditions: initial denaturing at 95 °C for 5 min, followed by 35 cycles of denaturing at 95 °C for 30 s, annealing at 54 °C for 30 s, and extension at 72 °C for 1 min; finalized by a 10-min elongation at 72 °C. The resulting amplicons were checked on a 0.8% (w/v) agarose gel and purified by precipitation with 3M potassium acetate (pH: 5) and isopropanol. Pure amplicons were quantified with the Qubit® 2.0 Fluorometer (Invitrogen, Carlsbad, CA, USA) and an equimolar pool of amplicons was prepared from all the samples.

### 16S rRNA gene sequencing and bioinformatic data analysis

A sequencing library was constructed with 100 ng of the equimolar pool by the amplicon fusion method (Ion Plus Fragment Library Kit, MAN0006846, Life Technologies). The library was quantified with the Agilent 2100 Bioanalizer (Agilent Technologies Inc, Palo Alto, California) prior to clonal amplification, and emulsion PCRs were carried out applying the Ion PGM Template OT2 400 kit as described in the user-guide (MAN0007218, Revision 3.0 Lifetechnologies) provided by the manufacturer. The library was sequenced in an Ion 318 Chip v2 on a Personal Genome Machine (PGM IonTorrent, Lifetechnologies) at Lifesequencing S.L (Lifesequencing, Valencia, Spain), using the Ion PGM Sequencing 400 kit following the manufacturer’s protocol (Publication Number MAN0007242, Revision 2.0, Lifetechnologies). Raw sequences obtained from the sequencing centre were processed with the MOTHUR software^28^. A summary of sequencing statistics is available in Supplementary Table 1. Short (<100 bp) and low quality (<Q10, <90% accuracy) reads were removed in a first step, and sequences were then assigned to samples based on barcode matches (allowing a maximum of 3 mismatches in primer search and 1 mismatch barcode search). The resulting sequences were trimmed by removing primer, barcode, and spacer sequences. All sequences were aligned to the ribosomal 16S Greengenes database using the kmer method (8-mers) for finding template sequences and the Needleman method for sequence alignment. Penalties for mismatch, gap opening, and gap extension were set as default (−1, −2, and −1, respectively). Finally, sequences were classified using BLAST searches against the same database. The similarity percentage cut off was set at 70%.

### Consortia identification and visualization

In order to identify microbial interactions, we used taxonomic data of samples from the ten machines. A method used a recently described multidimensional scaling and a biological significance filtering of the interactions^29^. Basically, the procedure was as follows: fluctuation scaling of replicas was validated by a Poisson distribution selection. Then, linear correlation coefficients were converted into distances and displayed by multidimensional scaling. Finally, a discrete Lotka-Volterra model with relative abundances^30^ was used to filter biologically significant interactions from the correlations identified in the last step.

## Additional Information

**How to cite this article**: Vilanova, C. *et al.* The coffee-machine bacteriome: biodiversity and colonisation of the wasted coffee tray leach. *Sci. Rep.* **5**, 17163; doi: 10.1038/srep17163 (2015).

## Supplementary Material

Supplementary Information

## Figures and Tables

**Table 1:** Nespresso machines sampled in this work.

Sample n°.	Machine model	Use*
1	*Essenza*	Communal (12 uses/day)
2	*Pixie*	Domestic (3 uses/day)
3	*Essenza*	Domestic (2 uses/day)
4	*CityZ* (capsule container)	Communal (10 uses/day)
5	*CityZ* (drip base)	
6	*Essenza*	Communal (20 uses/day)
7	*Inissia*	Domestic (4 uses/day)
8	*Essenza*	Communal (10 uses/day)
9	*Pixie*	Domestic (4 uses/day)
10	*Pixie*	Domestic (3 uses/day)
11–30	*Inissia* (Krups)	This work (3 uses/day)

^*^Frequency of use indicated in parenthesis as the average number of uses per day, as stated by the users of each machine.
