# Supplementary material for: The coffee-machine bacteriome: biodiversity and colonisation of the wasted coffee tray leach
Source: Sci Rep. 2015 Nov 23;5:17163. doi: 10.1038/srep17163 (PMC4655483; doi:10.1038/srep17163)
Supplement: Supplementary Information [file srep17163-s1.doc]

**Supplementary Table 1.** Summary of sequencing statistics from the 16S ribosomal RNA gene amplicon pool.

| Number of sequences obtained | 966,699 |
| --- | --- |
| Average length | 527.187 |
| Total Mb | 510.121 |
| Averag. number of sequences per sample | 17,901.833 |
| Number of sequences after trimming | 503,544 |
| Averag. length after trimming | 520.231 |
| Averag. number of sequences per sample after trimming | 15,258.909 |
| Number of bacterial genera (>0.01% abundance) | 59 |
